# Ligand Engineering of Inorganic Lead Halide Perovskite Quantum Dots toward High and Stable Photoluminescence

**DOI:** 10.3390/nano14141201

**Published:** 2024-07-15

**Authors:** Changbo Deng, Qiuping Huang, Zhengping Fu, Yalin Lu

**Affiliations:** 1 Department of Materials Science and Engineering, University of Science and Technology of China, Hefei 230026, China; 2 Hefei National Research Center for Physical Sciences at the Microscale, Anhui Laboratory of Advanced Photon Science and Technology, University of Science and Technology of China, Hefei 230026, China

**Keywords:** perovskite nanocrystals, CsPbX_3_, ligand modification, long-term stability, luminescence

## Abstract

The ligand engineering of inorganic lead halide perovskite quantum dots (PQDs) is an indispensable strategy to boost their photoluminescence stability, which is pivotal for optoelectronics applications. CsPbX_3_ (X = Cl, Br, I) PQDs exhibit exceptional optical properties, including high color purity and tunable bandgaps. Despite their promising characteristics, environmental sensitivity poses a challenge to their stability. This article reviews the solution-based synthesis methods with ligand engineering. It introduces the impact of factors like humidity, temperature, and light exposure on PQD’s instability, as well as in situ and post-synthesis ligand engineering strategies. The use of various ligands, including X- and L-type ligands, is reviewed for their effectiveness in enhancing stability and luminescence performance. Finally, the significant potential of ligand engineering for the broader application of PQDs in optoelectronic devices is also discussed.

## 1. Introduction

All-inorganic lead halide perovskite CsPbX_3_ (X = Cl, Br, I) quantum dots (PQDs) represent a significant class of functional materials that have been extensively studied and reported for their roles in optoelectronic conversion and as luminescent materials, particularly in areas such as light-emitting diodes (LEDs), PQD solar cells (PQD-SCs), and optical patterning [1,2,3,4,5,6]. PQDs are semiconductor nanocrystals (NCs) characterized by their three-dimensional confinement, which leads to the quantum confinement effect. PQDs possess excellent optical characteristics, such as a narrow full-width at half-maximum (FWHM), a high photoluminescence quantum yield (PLQY), and a tunable optical bandgap [7]. The narrow FWHM of all-inorganic lead halide PQDs results in high color purity, with a color gamut that can reach up to NTSC (National Television System Committee) 144% [8]. In addition, CsPbX_3_ PQDs exhibit a unique defect tolerance; the high defect tolerance implies that they can maintain a high PLQY even when some defects are introduced during the preparation of optoelectronic devices [9,10]. The tunable optical bandgap means that by altering the composition of halide atoms or adjusting the size or shape of the PQDs, it is possible to tune the emission wavelength across the entire visible spectral range (410–700 nm) [11]. Furthermore, all-inorganic PQDs demonstrate relatively higher thermal stability and photostability, compared to organic–inorganic hybrid perovskite (OIHP) QDs.

Since Kovalenko’s group first reported on PQDs in 2015, rapid progress has been made in the field [12]. In the same year, Zeng’s group first prepared blue, green, and yellow LEDs based on all-inorganic CsPbX_3_ (X = Cl, Br, I) PQDs [13]. In 2016, Swarnkar et al. fabricated stable black-phase CsPbI_3_ PQD films in ambient air, with the best device efficiency reaching 10.77% [14], and the currently certified efficiency has reached 19.1% [15]. Han’s group first prepared CsPbBr_3_ PQD-based photonic resistive random-access memory (RRAM), which offers higher reproducibility and stability compared to HOIP-based RRAM [16]. In 2020, Kim et al. used zwitterionic polymers as both ligands and matrices for CsPbBr_3_ PQDs and introduced the benzophenone structure onto the side chains of the polymers, achieving photolithographically patterned films [17]. It can be seen that PQDs show broad application prospects in various fields. However, there are still some demerits associated with PQDs that limit their practical applications. On one hand, the ionic crystal nature of PQDs makes them sensitive to external environmental conditions such as humidity, temperature, light exposure, and polar solvents, leading to structural instability. On the other hand, the traditional hot-injection protocol requires high temperatures and an inert gas environment, along with the use of long-chain ligands, such as oleic acid (OA) and oleylamine (OAm), to prevent aggregation of PQDs. To address these issues, a multitude of approaches have been proposed. Among these, surface ligand engineering has been widely adopted, which improves stability by addressing ligand detachment and halide migration and showing promising application prospects.

**Figure 1:**
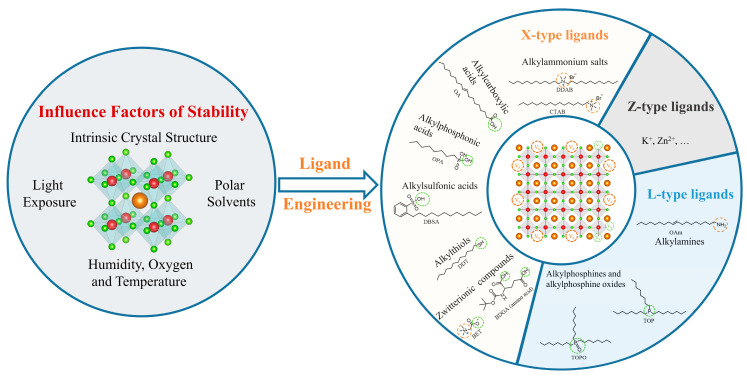
Schematic diagram of ligand engineering to overcome the instability factors of PQDs.

Here, we review the influence factors for PQDs’ instability, as well as various ligand engineering strategies to improve the stability of PQDs, which is outlined in Figure 1. In the first part, we briefly introduce the basic structure of PQDs and the factors that affect structural stability. In the following section, we address the synthesis methods of solution-based ligand exchange, focusing on in situ ligand engineering and post-synthesis ligand engineering. Additionally, various types of ligands, such as X- and L-type ligands, and their impact on stability are discussed.

## 2. The Basic Structure and Instability of CsPbX_3_ PQDs

### 2.1. Crystal Structure

**Figure 2:**
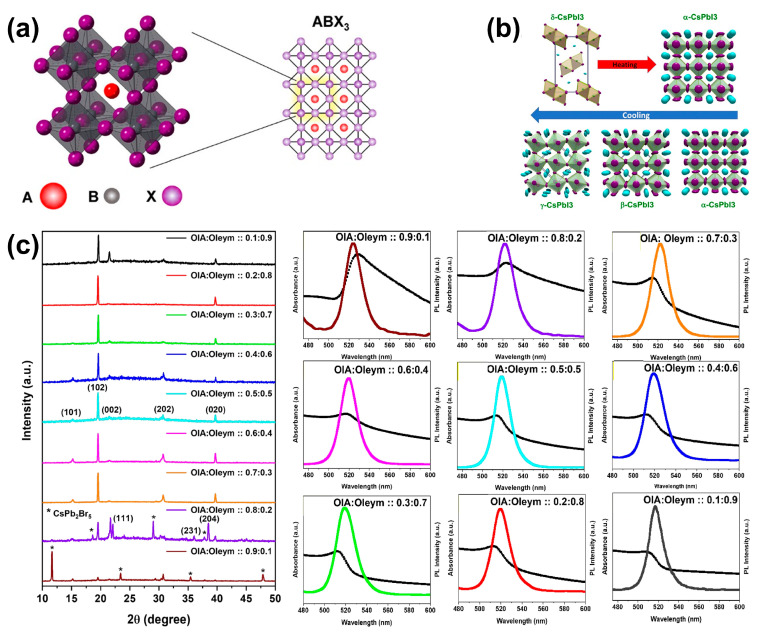
(**a**) The crystal structure of ABX_3_ (A = Cs; B = Pb; and X = Cl, Br, and I) perovskite. Reproduced with permission from ref. [22]. Copyright 2019, American Chemical Society. (**b**) Structural phase transitions of CsPbI_3_ perovskite. Reproduced with permission from ref. [23]. Copyright 2018, American Chemical Society. (**c**) XRD patterns (**left**) and UV–Vis absorbance (black) and PL spectra (colorful) (**right**) of CsPbBr_3_ PQDs synthesized with varying concentrations of OA (OIA) and OAm (Oleym) ligands. Reproduced with permission from ref. [24]. Copyright 2022, Springer Nature.

In the crystal structure of CsPbX_3_ perovskite, Cs^+^ occupies the corner positions of the lattice, Pb^2+^ is located at the center of the cube, X^−^ (X = Cl, Br, I) is at the center of six planes, and a Pb^2+^ forms an [PbX_6_] octahedral structure with the surrounding six X^−^ (Figure 1a). As shown in Figure 2b, CsPbX_3_ perovskites exhibit a rich variety of crystal structures, featuring four main forms: the cubic (α-), tetragonal (β-), orthorhombic (γ-), and non-perovskite orthorhombic (δ-) phases [18]. For the ABX_3_ perovskite structure, phase stability can be predicted through two important factors: the Goldschmidt tolerance factor (t) and the octahedral factor (μ) [19]. The tolerance factor is defined as follows [20]:

(1) $$t=\frac{r_{A}+r_{X}}{\sqrt{2}\left(r_{B}+r_{X}\right)}$$

where *r_A_*, *r_B_*, and *r_X_* represent the effective radii of the A-site, B-site, and X-site ions, respectively. For a conventional three-dimensional bulk perovskite, the t values typically range from 0.78 to 1.05. In an ideal cubic structure, the value is theoretically predicted to be 1. The other octahedral factor is defined as follows:

(2) $$\mu =\frac{r_{B}}{r_{X}}$$

It determines the stability of the octahedral structure, and the perovskite structure is stable when the value is between 0.44 and 0.9. For instance, the t and *μ* values for CsPbBr_3_ and CsPbI_3_ are (0.92 and 0.5) and (0.89 and 0.47), respectively [21]. It can be seen that both factors are related to ionic radii, thus highlighting the importance of controlling ionic radii to form a stable cubic structure.

### 2.2. Ligand

Ligands are molecules that can attach to the surface of PQDs, creating a coordination complex. These molecules are indispensable for the synthesis of PQDs, as they facilitate the nucleation and growth of the crystal and enable the production of PQDs with diverse shapes and sizes. In addition, ligands also serve to passivate surface defects on the PQDs. This defect passivation is essential as it significantly improves the luminescence performance and stability of the PQDs [25]. Regardless of the method used, whether it is the traditional hot-injection or ligand-assisted reprecipitation (LARP) method, long-chain alkyl-carboxylic acids and alkyl-amines, such as OA and OAm, are the most commonly used ligands. OA can chelate with lead atoms on the surface of PQDs, thereby inhibiting the aggregation of quantum dots, while OAm binds to halide ions on the PQDs’ surface through hydrogen bonding [26]. In the hot-injection method, they facilitate the dissolution of inorganic precursors in 1-octadecene (ODE) [27], and the structure and optoelectronic properties of PQDs can also be controlled by altering the ratio of OA and OAm (Figure 2c) [24]. However, the dynamic binding of ligands to the surface inevitably leads to the detachment of ligands, which in turn results in the instability of PQDs and affects their luminescent properties [28]. Therefore, using other ligands (such as multidentate ligands) to strengthen the binding with PQDs can more effectively passivate PQDs and improve their stability [29]. The presence of strong ligands can reduce the aggregation between PQDs and decrease the impact of the external environment on the PQD surface.

### 2.3. Influence Factors of Stability

The stability of metal halide PQD materials is a prerequisite for achieving stable photoluminescence. However, the instability of the crystal structure of PQDs, along with external environmental factors (humidity, temperature, light exposure, and polar solvents), limit their structural integrity and optical performance. In this section, we provide a detailed introduction to the factors that contribute to the instability of PQDs, as well as how these factors affect the luminescent properties of PQDs.

#### 2.3.1. Intrinsic Crystal Structure

**Figure 3:**
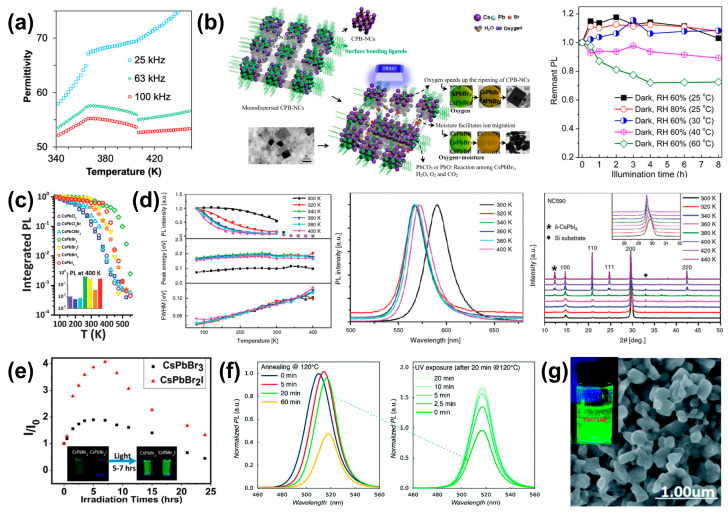
(**a**) Permittivity of (001) CsPbBr_3_ crystals measured at different frequencies on cooling. Reproduced with permission from ref. [32]. Copyright 2023, American Chemical Society. (**b**) (**Left**) Schematics of the possible degradation pathways of the CsPbBr_3_ film under the effects of humidity and oxygen. (**Right**) The variation in PL intensity of CsPbBr_3_ films stored in the dark at different relative humidity (RH) levels and temperatures. Reproduced with permission from ref. [34]. Copyright 2017 American Chemical Society. (**c**) The integrated PL of CsPbX_3_ PQD samples varies with temperature. The inset bar plot shows the fraction of emission (relative to 80 K) at 400 K. Reproduced with permission from ref. [35]. Copyright 2017, Wiley-VCH. (**d**) PL intensities, peak energies, linewidths (**left**), normalized PL spectra (**middle**), and XRD patterns (**right**) of the CsPbBr_2_I PQD films annealed at various temperatures. The inset in the right image shows a magnified view of (200) crystal-plane diffraction peaks. Reproduced with permission from ref. [36]. Copyright 2019, CSIRO. (**e**) Plot of relative emission intensity (I/I_0_) of the PQDs versus light irradiation time. Inset: photographs of emission enhancement on photoactivation. Reproduced with permission from ref. [37]. Copyright 2016, American Chemical Society. (**f**) PL emission spectra of CsPbBr_3_ PQD films for various annealing times (**left**) and UV exposure times (**right**). Reproduced with permission from ref. [38]. Copyright 2018, The Royal Society of Chemistry. (**g**) Field emission scanning electron microscopy (FE-SEM) image and photograph (inset) of OA/OAm-CsPbBr_3_ PQDs after adding TMEDA. Reproduced under terms of the CC-BY 4.0 license. Ref. [39] Copyright 2021, the authors. Published by the Royal Society of Chemistry.

As mentioned above, CsPbX_3_ PQDs primarily exhibit four crystal structures: the cubic (α-), tetragonal (β-), orthorhombic (γ-), and non-perovskite orthorhombic (δ-) phases. These phases can be reversibly interconverted by changing temperature [30]. During the cooling process of CsPbCl_3_, the phase transition from the α-phase to the β-phase occurs at 320 K; the transition from the β-phase to the γ-phase occurs at 315 K; and finally, the transformation to the δ-phase (monoclinic) takes place at 310 K. It can be observed that the phase transitions of CsPbCl_3_ all occur at relatively low temperatures, which is the reason for its instability at room temperature [31]. In CsPbBr_3_, there are mainly three crystal structures, with the α-phase nanocrystals having the highest luminescence efficiency. Bari et al. demonstrated that CsPbBr_3_ crystals undergo two phase transitions during cooling; the first transition from the α-phase to the β-phase occurs at 403 K, and the other is the transition from the β-phase to the γ-phase at 361 K (Figure 3a) [32]. Therefore, the cubic structure of CsPbBr_3_ exists at high temperatures [33]. In CsPbI_3_, the phase transition from the α-phase (black phase) to the β-phase occurs at 539 K; the transition from the β-phase to the γ-phase occurs at 425 K; and finally, the transformation to the δ-phase (non-perovskite structure, yellow phase) takes place at room temperature [31]. Furthermore, the γ-phase and δ-phase are stable at room temperature, while the α-phase and β-phase are stable at high temperatures. CsPbCl_3_, CsPbBr_3_, and CsPbI_3_ are utilized for emitting blue, green, and red light, respectively, with the entire visible spectrum tunable through precise adjustments in the ratios of the halides.

#### 2.3.2. Effects of Humidity, Oxygen, and Temperature

In the actual process of fabricating PQD-based luminescent devices, humidity and oxygen are often simultaneously present. Due to the ionic properties of PQDs, they are prone to degradation in environments that are humid and/or contain oxygen, which in turn deteriorates their luminescent stability (Figure 3b left) [34]. Water molecules penetrate into the inner inorganic framework of PQDs, and in the presence of oxygen, CsPbX_3_ is likely to form lead hydroxides (Pb(OH)_2_) and lead oxide (PbO), leading to the complete degradation of PQDs [40].

2CsPbX_3_·H_2_O + 0.5O_2_ + CO_2_ → 2CsX + PbCO_3_ + Pb(OH)_2_ + 2HX + X_2_ (3)

Pb(OH)_2_ → PbO + H_2_O (4)

The decomposition under thermal stress and thermal-induced PL quenching that occur at high temperatures are the main factors affecting the PL properties of PQDs (Figure 3b right). On one hand, degradation due to humidity and oxygen is accelerated and amplified at high temperatures, decreasing the PL intensity. On the other hand, the thermal-induced PL quenching results from the aggregation of PQDs will aggravate with increasing temperature or time [41,42,43,44]. Diroll et al. studied the PL intensities of CsPbX_3_ as the temperature increased from 80 K to 500 K (Figure 3c). They found that the PL of CsPbCl_3_ began to drop sharply from 300 K, with the most significant reduction in intensity, while CsPbBr_3_ and CsPbI_3_ showed a sharply declined PL intensity near 375 K, with CsPbBr_3_ retaining the highest PL intensity at 500 K [35]. Upon prolonged heating temperature or time, significant changes in the PL intensity, peak position, or crystal structure of CsPbX_3_ PQDs may occur, characterized by a substantially decreased intensity, an obvious blue-shift PL peak, and changes in XRD patterns (Figure 3d) [36].

#### 2.3.3. Effects of Light Exposure

Continuous illumination (photostability) can also affect the luminescence performance and long-term stability of PQDs. At the beginning of light exposure, the PL intensity and transient decay time will increase, which is known as “photoactivation effect”. Seth et al. found that the PL intensity and average lifetimes of CsPbBr_3_ and CsPbBr_2_I both increased under light illumination within 5–7 h, with the PL intensity increasing by 2 times and 4.5 times, respectively (Figure 3e). They believe this may be due to the structural reorganization and the filling of trap states in PQDs. Meanwhile, this phenomenon was more prominently in the CsPbBr_2_I sample, characterized with distorted crystal structure [37]. However, prolonged light exposure will cause a slow decline in PL intensity and a red-shift in the spectrum with the aggregation of PQDs into larger nanocrystals due to ligand desorption and crystal regrowth [45]. Moyen et al. utilized the photoactivation effect in LED devices by removing ligands that hinder charge carrier injection through annealing and then exposing them to ultraviolet (UV) light in air to enhance the PL of the PQD film (Figure 3f) [38].

#### 2.3.4. Polar Solvents

In previous reports, PQDs were often dispersed in nonpolar solvents, such as toluene, hexane, or octane, due to the poor stability in polar solvents [46]. Sun et al. found that polar solvents will accelerate the detachment of ligand molecules from the surface of PQDs, which in turn forms new defects and reduces the intensity. The interesting part is that although tetramethylethylenediamine (TMEDA) also breaks the original equilibrium state of the ligands, the effective luminescence of PQDs is not completely destroyed, and there is still fluorescence several months later (Figure 3g) [39]. Kovalenko’s group designed and synthesized a series of phospholipid zwitterionic ligands, improving the long-term colloidal stability and compatibility of PQDs in various solvents by altering the structure of the ligand’s tail group [47].

As discussed above, under the influence of various factors, ligand detachment will lead to the chemical instability, particle agglomeration, or degradation of PQDs, causing a decline in PLQY [48]. Although light exposure can bring about a short-term improvement, prolonged exposure to light will still lead to PL quenching. Therefore, it is very necessary to design new ligands to replace traditional ones for a more stable luminescence of PQDs.

## 3. Ligand Engineering

### 3.1. Synthesis Methods

#### 3.1.1. In Situ Ligand Engineering

In the two commonly used synthetic methods (hot-injection and LARP), OA and OAm are the most frequently used ligands, but they are prone to dissociation and lead to PL quenching. In situ and post-synthesis ligand engineering have been employed to enhance the binding of ligands to solution-based PQDs, achieving good environmental stability and high PLQY. In these two ligand engineering methods, the former is simpler and easier to operate during the whole synthesis process, without the need for further treatment and purification steps.

**Figure 4:**
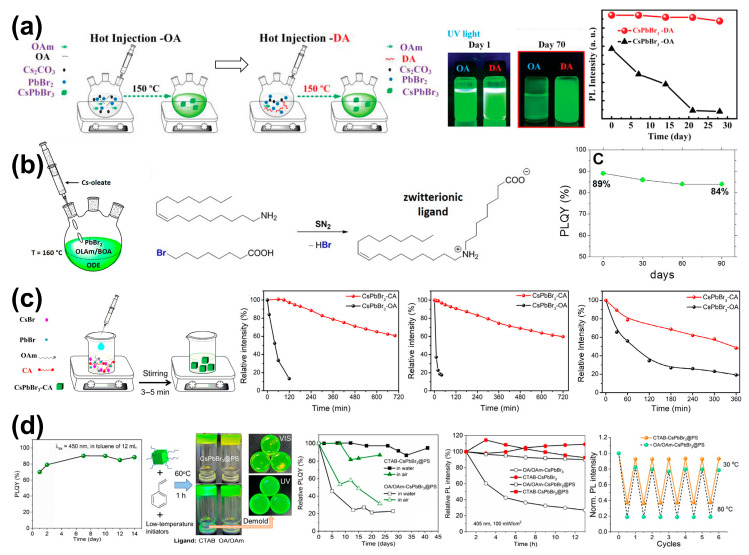
(**a**) (**Left**) Schematic diagram of the synthesis of CsPbBr_3_ PQDs using DA as a substitute for OA via in situ ligand engineering. (**Middle**) Photographs of PQD colloidal solution with OA/DA under UV light stored in air. (**Right**) PL intensity variation of PQD films in air. Reproduced with permission from ref. [49]. Copyright 2019, Wiley-VCH. (**b**) (**Left**) Schematic diagram of the synthetic approach for CsPbBr_3_ PQDs with OAm (which is referred to as OLAm in the figure) and BOA. (**Middle**) In situ formation of zwitterionic ligands by the reaction of native ligands. (**Right**) The variation in PLQY of CsPbBr_3_ PQDs under ambient conditions. Reproduced under terms of the CC-BY 4.0 license. Ref. [50] Copyright 2022, the authors. Published by American Chemical Society. (**c**) (**Left**) Schematic illustration of the preparation of CsPbBr_3_-CA PQDs. (**Right**) Relative intensity variations of CsPbBr_3_-OA and CsPbBr_3_-CA upon exposure to water, ethanol, and continuous UV light, respectively. Reproduced with permission from ref. [51]. Copyright 2021, Springer Nature. (**d**) (**Left**) The evolution of the PLQY for CTAB-CsPbBr_3_ PQDs stored under ambient conditions. (**Middle**) Photographs of the CsPbBr_3_@PS composite films with CTAB or OA/OAm as ligand(s). (**Right**) The evolution of PLQY (or PL intensity) of uncoated and coated CsPbBr_3_ PQDs is examined under various conditions, including in water and air, under light irradiation, and during thermal cycles. Reproduced with permission from ref. [52]. Copyright 2022, American Chemical Society.

In the hot-injection method, Yan et al. effectively improved the colloidal solution and film luminescence stability of CsPbBr_3_ PQDs by introducing 2-hexyldecanoic acid (DA) with two short branched chains to replace OA for surface modification during the synthesis process, as shown in Figure 4a left. The PQDs did not show aggregation phenomena even after being stored in the air for over 70 days (Figure 4a middle), and the film still maintained 94.3% of its initial PL intensity after 28 days (Figure 4a right) [49]. Grisorio et al. introduced an extra halide source (8-bromooctanoic acid, BOA) to form a zwitterionic ligand in situ, and the PLQY of the preserved sample decreased from the initial 89% to 84% after 90 days, which was much more stable than that of the untreated CsPbBr_3_ PQDs, of which the PLQY decreased from the initial 95% to less than 30% after 90 days (Figure 4b) [50].

In the LARP method, double-terminal ligand 4,4′-Azobis(4-cyanovaleric acid) (CA) is introduced to replace OA in the synthesis of CsPbBr_3_ PQDs (Figure 4c left). The resulting PQDs exhibit a PLQY of 72% and also have better stability, with the PL intensity maintaining 80%, 75%, and 50% of its initial intensity after being immersed in water and ethanol and under continuous illumination for 6 h, respectively (Figure 4c right) [51]. Cai et al. introduced a bromide-rich ligand, cetyltrimethylammonium bromide (CTAB), to replace OA and OAm, and the resulting CsPbBr_3_ PQDs had a PLQY of 70% (superior to the 64% obtained with OA and OAm as ligands). The stored CsPbBr_3_ PQDs could achieve a PLQY of up to 90% after 7 days (Figure 4d left); CsPbBrI_2_ PQDs could achieve a PLQY of up to 65%. By further using polystyrene (PS) for polymer encapsulation of the PQDs, their stability against water, heat, and light illumination was improved (Figure 4d middle and right) [52].

#### 3.1.2. Post-Synthesis Ligand Engineering

**Figure 5:**
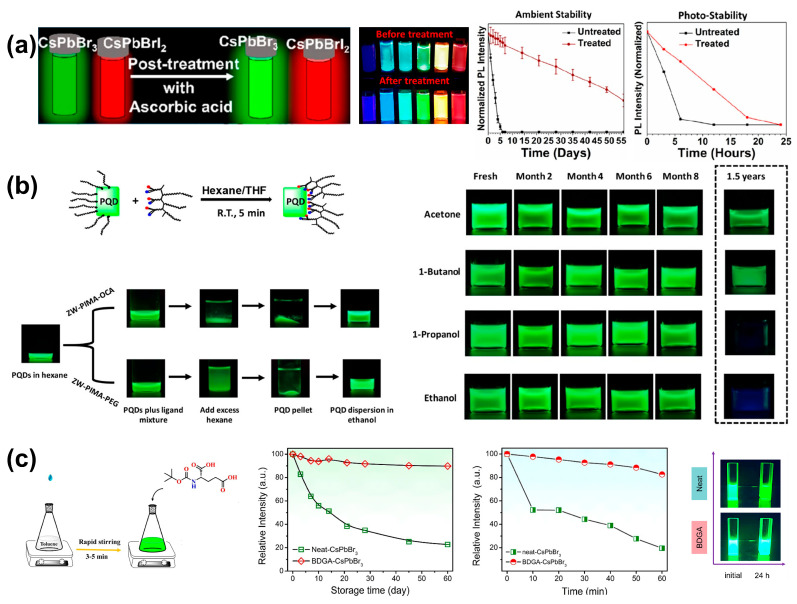
(**a**) (**Left**) Schematic diagram of post-treatment with AA for CsPbX_3_ PQDs. (**Middle**) Photographs of CsPbX_3_ PQD colloidal solutions before and after treatment under UV illumination. (**Right**) The evolution of the PL intensity for untreated and treated PQDs stored under ambient conditions or continuous UV light, respectively. Reproduced with permission from ref. [53]. Copyright 2022, American Chemical Society. (**b**) (**Left**) Schematic diagram (**top**) and photographs (**bottom**) of the post-treatment process. (**Right**) Fluorescence images of ZW-PIMA-OCA−PQD dispersions in various polar solvents. Reproduced with permission from ref. [54]. Copyright 2020, American Chemical Society. (**c**) (**Left**) Schematic representation of the ligand exchange process with BDGA. (**Middle**) The evolution of the PL intensity for neat- and BDGA-CsPbBr_3_ stored under ambient conditions and continuous heating, respectively. (**Right**) Photographs of neat- and BDGA-CsPbBr_3_ colloidal solution before and after under continuous UV light. Reproduced with permission from ref. [55]. Copyright 2022, Elsevier B.V.

In the hot-injection method, Mishra’s group improved the PL intensity and stability of CsPbX_3_ by using ascorbic acid (AA) as a surface capping ligand for post-treatment (Figure 5a left); the PLQY of CsPbBr_3_, CsPb(Br/I)_3_, CsPbCl_1_._5_Br_1_._5_, and CsPbClBr_2_ were increased from 72% to 99%, from 22–55% to over 95%, from 12% to 22%, and from 41% to 51%, respectively (Figure 5a middle). In particular, the PLQY of CsPbI_3_ PQDs has been improved from 51% to over 95%, with no phase change observed even after 55 days in an ambient environment, also exhibiting enhanced stability under UV illumination, with 76.7% of initial PL intensity retained, compared to completely lost in 4 h (Figure 5a right) [53]. Wang et al. synthesized two novel zwitterionic polymers (ZW-PIMA-OCA and ZW-PIMA-PEG) for ligand exchange, which enable high-affinity coordination with the surfaces of PQDs due to the presence of multiple sulfobetaine groups on each ligand. After ligand exchange with ZW-PIMA-OCA and ZW-PIMA-PEG, the PLQY was enhanced from 55–60% to 65–75% and 70–80%, respectively (Figure 5b left). Moreover, the ZW-PIMA-OCA-PQDs remained bright and homogeneous after storage in acetone and 1-butanol for 1.5 years, demonstrating excellent long-term stability (Figure 5b right) [54].

In the LARP method, there have been relatively fewer reports on ligand exchange through post-synthesis ligand engineering compared to the hot-injection method. Based on the use of CA as a ligand to replace OA, Zhang et al. performed ligand exchange with a multidentate ligand Boc-D-Glutamic acid (BDGA), achieving a PLQY close to 100% (Figure 5c left). Additionally, the long-term storage stability, which is 90% versus 22% of the initial PL after 60 days; thermal stability, which is over 80% versus 20% retained after heating at 60 °C for 60 min; and photostability, which is 95% versus 40% under constant UV radiation for 24 h, were all improved (Figure 5c middle and right) [55].

### 3.2. Classification of Ligands

To enhance the luminescence stability of PQDs, a variety of ligand engineering strategies have been developed to strengthen the coordination interaction between ligands and PQDs. The binding modes between ligands and metals can be categorized differently according to the Covalent Bond Classification (CBC) [56]. Based on the number of electrons donated by the ligands to the binding bond, they are divided into L-, X-, and Z-type ligands, which provide 2, 1, and 0 electrons to the metal, respectively. L-type ligands, which are Lewis bases, can interact with the surface metal cations of the PQDs, with examples including alkylamines (NH_2_R) and alkylphosphine oxides (PR_3_). X-type ligands donate electron to the surface cations (Cs^+^ or Pb^2+^) and halide anions, and they are diverse in type, mainly including alkylammonium salts (R_4_N^+^, R can be H), alkylcarboxylic acids (RCOO^−^), alkylphosphonic acids (RPO(OH)O^−^), alkyl sulfonic acids (RSO^3−^), alkylthiols (RSH), and zwitterionic compounds. Z-type ligands are Lewis acids that coordinate with the halide anions on the surface of PQDs [57]. X- and L-type ligands are predominantly utilized in surface engineering. Therefore, this review concentrates on the application of these two ligand types.

### 3.3. X-Type Ligands

#### 3.3.1. Alkylammonium Salts

OA can protonate OAm, causing the detachment of ligands from the surface of PQDs and the formation of vacancies at the A- and X-site. However, ammonium salts have both cations and anions, and the cations can prevent the protons provided by the acid and passivate the A-site vacancies, while the anions can passivate the vacancies at the X-site. Consequently, the method of modulating PL properties by altering the structure of the ammonium salt side chains is highly favored [58,59].

**Figure 6:**
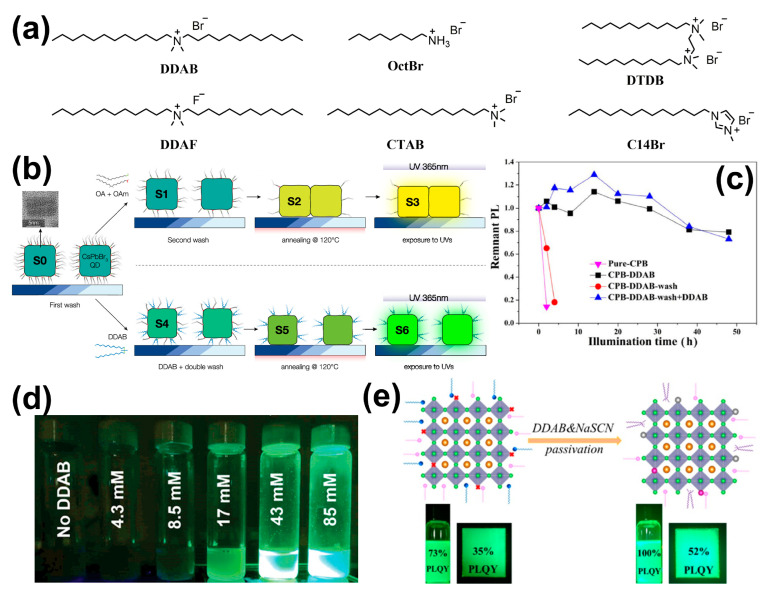
(**a**) The molecular chemical structures of DDAB and other alkylammonium salts. (**b**) Schematic diagrams of various treatments applied to CsPbBr_3_ PQD films with OA/OAm or DDAB as ligands. Reproduced with permission from ref. [60]. Copyright 2018, American Chemical Society. (**c**) The photostability of CsPbBr_3_ (CPB) PQDs. Reproduced with permission from ref. [61]. Copyright 2019, Springer Nature. (**d**) Photographs of PQDs dispersed in fully methanol/butanol environments after 5040 h. Reproduced with permission from ref. [62]. Copyright 2023, the authors, published by Wiley-VCH. (**e**) Surface passivation of CsPbBr_3_ PQD colloidal solution and film by treatment with DDAB and NaSCN. Reproduced with permission from ref. [63]. Copyright 2019, American Chemical Society.

Among all ammonium salt ligands, dodecyltrimethylammonium bromide (DDAB) is most commonly utilized, and the molecular chemical structures of DDAB and other alkylammonium salts are shown in Figure 6a [60,61,62,63,64,65,66]. It will effectively prevent the detachment of ligands during the purification process and thus enhance the washing stability (Figure 6b) [60,61]. Moreover, it also significantly improves the photostability of PQDs (Figure 6c) [61,65]. Post-treatment with DDAB can passivate surface defects and induce PQD aggregation, which impedes the permeation of alcohol molecules and reduces the density of halide deficient sites. Specifically, CsPbBr_3_ PQDs can achieve a stability of up to 7 months with a PLQY approaching unity in fully methanol/butanol environments, which greatly enhances the long-term stability in polar solvent environments (Figure 6d) [62]. Furthermore, DDAB is often used in combination with other ligands to form hybrid ligands, which collectively improve PL performance and stability. Post-treatment with DDAB and sodium thiocyanate (NaSCN) can fully passivate the surface of CsPbBr_3_ PQDs, increasing the PLQY of the PQD colloidal solution from 73% to 100% and of PQD film from 35% to 52%, respectively (Figure 6e). This is mainly attributed to the simultaneous filling of bromine vacancies of PQDs by Br^−^ and SCN^−^, which reduces the surface defect density and decreases the sites for non-radiative recombination [63]. A hybrid ligand composed of DDAB and ZnBr_2_ can effectively passivate the surface defects formed by halogens, simultaneously enhancing the PLQY from 70% to 95% as well as the long-term storage, UV exposure, washing, and thermal stability. The reason is that DDAB is permanently charged fully, which suppresses the acid–base reactions between ligands; simultaneously, the hybrid ligand can firmly anchor on the surface, passivating the surface defects [67]. Post-treatment with didodecyldimethylammonium fluoride (DDAF) can completely suppress the thermal quenching of CsPbBr_3_ PQDs, maintaining a PLQY of 90% even when the temperature rises to 373 K, effectively improving the thermal stability of the PQDs. Fluoride ions not only occupy the bromine vacancies on the surface of PQDs but also replace the shallow bromide ions, forming fluorine-rich surfaces. Compared to the inner core, these surfaces have a wider bandgap, which suppresses carrier trapping and enhances thermal stability [68]. Therefore, DDAB has been demonstrated to be an effective ligand for achieving high and stable PL in PQDs. In addition to DDAB, other ammonium salt ligands such as octylammonium hydrobromide (OctBr) [69], cetyltrimethylammonium bromide (CTAB) [52], N1.N2-didodecyl-N1. N1.N2.N2-tetramethylethane-1,2-diaminium bromide (DTDB) [70], and 1-tetradecyl-3-methylimidazolium bromide (C14Br) [71] also play extremely important roles in enhancing the PL performance and stability of PQDs.

#### 3.3.2. Alkylcarboxylic Acids

**Figure 7:**
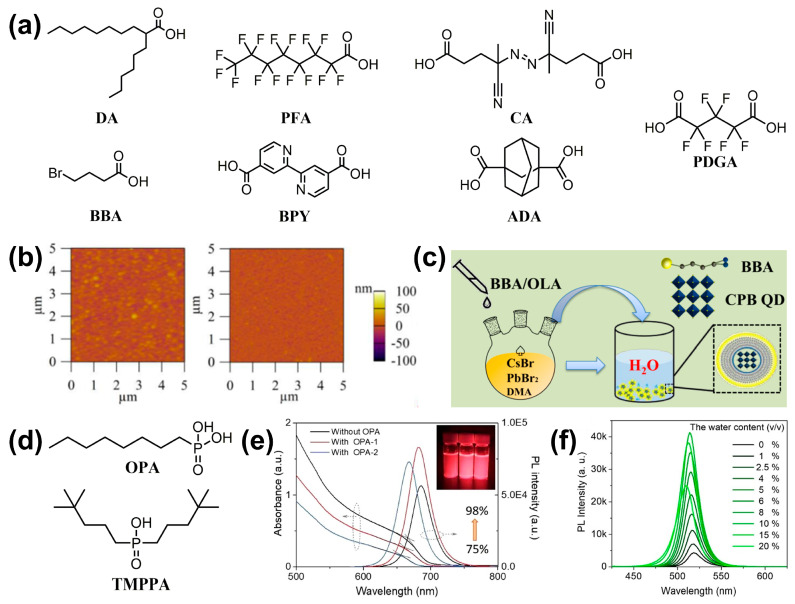
(**a**) The molecular chemical structures of DA and other alkylcarboxylic acids. (**b**) AFM images of CsPbBr_3_-OA (**left**)) and CsPbBr_3_-DA (**right**) PQD films. Reproduced with permission from ref. [49]. Copyright 2019, Wiley-VCH. (**c**) Schematic diagram of the synthetic process of CsPbBr_3_ PQDs by the LARP method. Reproduced with permission from ref. [73]. Copyright 2022, Wiley-VCH. (**d**) The molecular chemical structures of two alkylphosphonic acids. (**e**) UV–vis absorption and PL spectra of CsPbI_3_ PQDs with different amount of OPA. Reproduced with permission from ref. [79]. Copyright 2020, Elsevier B.V. (**f**) PL spectra of CsPbBr_3_ PQDs post-treated with different amounts of water. Reproduced with permission from ref. [80]. Copyright 2023, Elsevier B.V.

As mentioned above, ammonium salts are used to passivate the surface defects of PQDs by replacing traditional OAm surface ligands, thereby enhancing their stability. Alkylcarboxylic acid ligands serve the same purpose by fully or partially replacing conventional OA ligands, binding to one or more lead sites. In the hot-injection method, the introduction of 2-hexyldecanoic acid (DA, Figure 7a) as a substitute for OA has led to the production of PQD films with higher crystallinity and smoother morphology (Figure 7b) [49], which also exhibit improved luminescent performance and stability when applied in LEDs [72]. In a method that can be viewed as an improved LARP method, 4-bromo-butyric acid (BBA) is used to replace OA, which facilitate in situ crystallization by binding to Pb^2+^ on the surface of PQDs through its carboxylic group (Figure 7c). A high PLQY of 86.4% can be obtained even when using water as a nonpolar solvent, and the resulting PQDs possess excellent stability under high humidity conditions. The synergistic effect of the carboxyl group in BBA and the amine cation in OAm forms a hydrophobic shell, thereby enhancing the decomposition resistance in polar solvents, including aqueous solution [73]. Furthermore, BBA can also act as a source of Br^−^ ions to enable anion exchange in CsPbI_3_ PQDs, allowing for fine-tuning of their emission color and bandgap [74]. The in situ partial replacement of OA with perfluorooctanoic acid (PFA) inhibits the transition of CsPbI_3_ PQDs to the δ-phase, maintaining the α-phase for up to 120 days. Due to the superior hydrophobic nature of PFA, it enhances the moisture resistance of the PQDs [75]. Additionally, the introduction of double-terminal ligands that bind to two lead sites, such as 2,2′-bipyridine-4,4′-dicarboxylic acid (BPY) [76], 4,4′-Azobis(4-cyanovaleric acid) (CA) [51], 1,3-adamantanedicarboxylic acid (ADA) [77], and perfluoroglutaric acid (PFGA) [78], has been shown to achieve high PLQY and high stability in CsPbX_3_ PQDs.

#### 3.3.3. Alkylphosphonic Acids

Alkylphosphonic acid ligands can passivate the uncoordinated Pb atoms in CsPbX_3_ PQDs, enhancing their chemical durability against the invasion of polar solvents [81]. Octylphosphonic acid (OPA, Figure 7d) passivates the surface defects of CsPbBr_3_ PQDs by forming a hydrogen bonding network through P=O and P-OH groups between ligands, which can also improve the PLQY (close to unity) and solution stability of CsPbI_3_ PQDs (Figure 7e) [79]. Similarly, bis(2,4,4-trimethylpentyl)-phosphinic acid (TMPPA) also enhances the PLQY and stability by forming inter-ligand hydrogen bonds and subsequently reducing surface defects through a water treatment process (Figure 7f). Thus, this hydrogen bond network has enhanced the binding strength between the PQDs and ligands, resisting the destructive effects of polar solvents [80].

#### 3.3.4. Alkylsulfonic Acids

**Figure 8:**
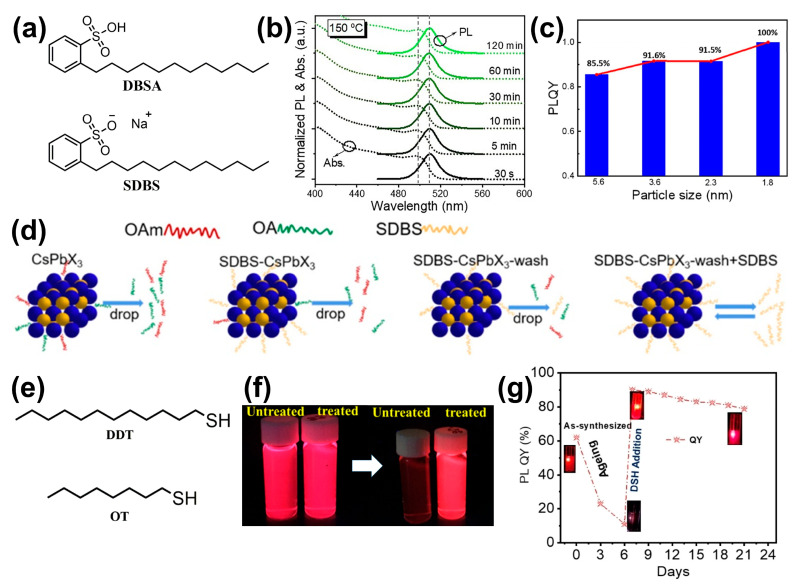
(**a**) The molecular chemical structures of DBSA and SDBS. (**b**) UV–Vis absorption and PL spectra of DBSA-QDs with different reaction times. Reproduced with permission from ref. [83]. Copyright 2022, Elsevier B.V. (**c**) PLQY of CsPbBr_3_ PQDs with different sizes. Reproduced with permission from ref. [84]. Copyright 2023, Elsevier B.V. (**d**) Dynamic diagram of SDBS showing strong binding interaction with the surface of PQDs. Reproduced with permission from ref. [85]. Copyright 2023, American Chemical Society. (**e**) The molecular chemical structures of DDT and OT. (**f**) Photographs shows the photostability of treated CsPbI_3_ after 24h under UV light. Reproduced with permission from ref. [86]. Copyright 2019, The Korean Society of Industrial and Engineering Chemistry. Published by Elsevier B.V. (**g**) The evolution of the PLQY for CsPbI_3_ PQDs, with the addition of DDT (which is referred to as DSH in the figure) after day 6. Reproduced with permission from ref. [87]. Copyright 2023, Wiley-VCH.

Alkylsulfonic acids form a stable binding state with lead ions exposed on the surface of PQDs through their strong ionic sulfonate heads, which can eliminate non-radiative recombination, addressing issues related to purification and storage [82]. Among them, dodecylbenzene sulfonic acid (DBSA) and its sodium salt (SDBS) are the most typical, and their molecular structures are shown in Figure 8a [82,83,84,85]. CsPbBr_3_ PQDs prepared with DBSA as a ligand not only prevent the formation of impurity phases Cs_4_PbBr_6_ and CsPb_2_Br_5_ at high temperatures but also extend the reaction time window from 30 s to 2 h (Figure 8b) due to the intense interaction between the sulfonate groups and lead ions [83]. Additionally, the combination of DBSA with cesium can serve as a precursor, and ultra-small sized CsPbBr_3_ PQDs with a size of 1.8 nm and a PLQY as high as 100% can be obtained by adjusting the amount of DBSA (Figure 8c). DBSA possesses strong electron-attracting properties, which can effectively passivate the surface defects of PQDs, thereby enhancing their optical performance [84]. SDBS also improves the issue of poor stability caused by the loss of amine ligands through its strong binding interaction with lead ions (Figure 8d), enhancing both the water stability and the washing stability [85].

#### 3.3.5. Alkylthiols

Alkylthiols modify PQDs by forming Pb-S bonds with them. When 1-dodecanethiol (DDT, Figure 8e) is used for ligand exchange immediately after the synthesis of CsPbI_3_ PQDs, it can significantly enhance their photostability under UV light exposure (Figure 8f). The high affinity of DDT for Pb^2+^ enables it to attach to the surface of the PQDs. The Pb-S bond possesses a higher dissociation energy compared to the Pb-O and Pb-I bonds, which aids in protecting the surface of the PQDs and prevents photo-induced structural changes [86]. When DDT is added to degraded CsPbI_3_ PQD dispersion, it can rapidly restore their crystal structure and luminescence (Figure 8g), thereby enhancing their PL intensity and ambient stability. The DDT ligands strongly adsorb onto the PQDs via Pb-S bonds, transforming the distorted octahedral structure of the aged sample into cubic CsPbI_3_ and enhances the environmental stability for several weeks through the S-capping mechanism [87]. The synergistic effect of 1-octanethiol (OT) with other ligands can improve PL intensity as well as photostability [88].

#### 3.3.6. Zwitterionic Compounds

**Figure 9:**
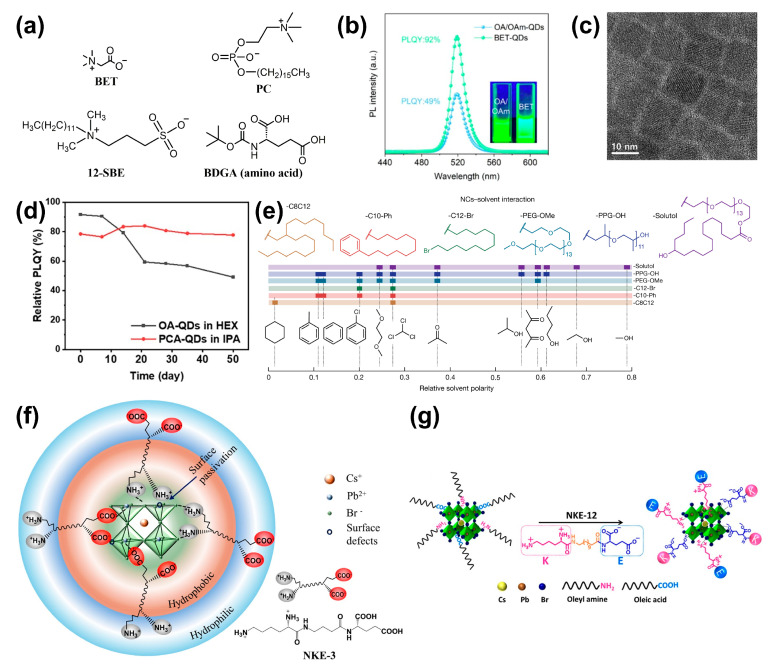
(**a**) The molecular chemical structures of BET and other zwitterionic compounds. (**b**) PL spectra and fluorescence images (inset) of OA/OAm- and BET-QDs. Reproduced with permission from ref. [89]. Copyright 2022, Elsevier B.V. (**c**) S-BET-treated PQDs maintained their original structure after thermal annealing. Reproduced with permission from ref. [90]. Copyright 2023, American Chemical Society. (**d**) PQDs with BET-based polymeric zwitterionic (PCA) as ligands exhibit enhanced stability in isopropanol (IPA). Reproduced with permission from ref. [93]. Copyright 2023, Elsevier B.V. (**e**) Different tails enable long-term stability of PQDs in specific solvents. Reproduced with permission from ref. [47]. Copyright 2023, the authors. (**f**) The schematic shows that the amino acid (NKE-3) binding to the surface of PQDs. Reproduced with permission from ref. [94]. Copyright 2023, American Chemical Society. (**g**) The schematic shows the replacement of OAm and OAc with the amino acid (NKE-12) on the surface of PQDs. Reproduced with permission from ref. [95]. Copyright 2023, American Chemical Society.

In the literature reported, a variety of zwitterionic ligands are utilized, which mainly include betaines (BET) [89], sulfobetaines (S-BET) [90], phosphocholine (PC) [91], amino acids [92], etc. These ligands (molecular structures as illustrated in the Figure 9a) can stably bind to the surface of PQDs through a chelation effect, significantly enhancing the durability and stability of PQDs. BET anchors to the surface of PQDs through the −COO^−^ and −N^+^(CH_3_)_3_ groups, markedly reducing the surface defects of CsPbBr_3_ PQDs, achieving a high PLQY of 92%, and improving resistance to external environments (Figure 9b). The enhancement in stability is attributed to the positively charged quaternary ammonium groups that are hanging over the surface of the PQDs, forming a positively charged outer layer that effectively prevents the aggregation of PQDs [89]. S-BET, due to its strong ligand binding affinity, also exhibits extraordinary stability after heat treatment [90]. Polymeric zwitterionic ligands prepared based on BET and S-BET can effectively enhance the phase stability of PQDs in polar solvents (Figure 5b right and Figure 9c) [54,93]. PC-type ligands enable CsPbI_3_ PQDs to maintain phase stability in air for at least six months [91]; long-term stability of PQDs in specific solvents can be endowed by altering the structure of their ligand tails (Figure 9d) [47]. Amino acids, as a type of zwitterionic ligand, are more likely to bind to the surface of PQDs through polycarboxylic structures to achieve surface defect passivation and enhancement of colloidal stability (Figure 9e,f) [55,94,95].

### 3.4. L-Type Ligands

#### 3.4.1. Alkylamines

**Figure 10:**
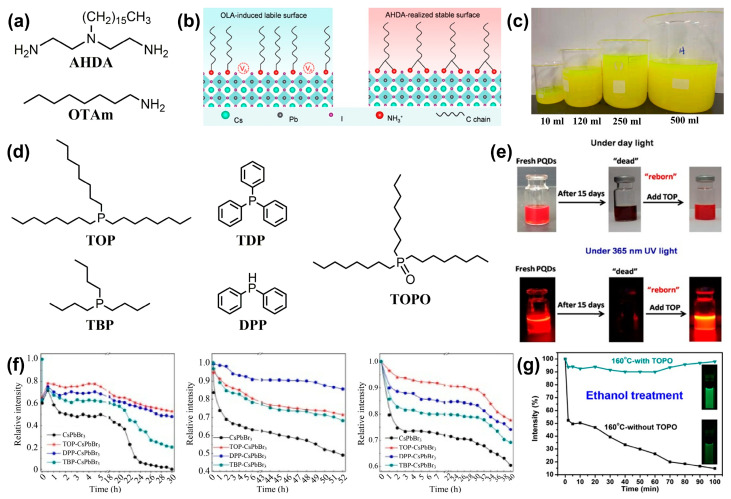
(**a**) The molecular chemical structures of AHDA and OTAm. (**b**) The ligand AHDA exhibits a stable interaction with the surface of PQDs. Reproduced with permission from ref. [96]. Copyright 2022, American Chemical Society. (**c**) Photographs representing the preparation of PQDs at various magnification scales. Reproduced with permission from ref. [97]. Copyright 2022, Elsevier B.V. (**d**) The molecular chemical structures of TOP and other alkylphosphines and alkylphosphine oxides. (**e**) Aged PQDs recover luminescence emission with the addition of TOP. Reproduced with permission from ref. [98]. Copyright 2018, American Chemical Society. (**f**) The evolution of the PL intensities for CsPbBr_3_ PQDs, TOP, DPP, or TBP modified CsPbBr_3_ PQDs upon the introduction of ethanol, water, or UV light irradiation. Reproduced with permission from ref. [99]. Copyright 2019, Springer Nature. (**g**) Stability of CsPbBr_3_ PQDs with or without TOPO against ethanol treatment. Reproduced with permission from ref. [100]. Copyright 2017, American Chemical Society.

Similar to the use of alkylcarboxylic acid ligands to replace OA, new L-type ligands are commonly used to substitute OAm. The synthesis of CsPbI_3_ PQDs is achieved using the polyamine chelate ligand N′-(2-aminoethyl)-N′-hexadecyl ethane-1,2-diamine (AHDA, Figure 10a) as a substitute for OAm. The protonated AHDA can anchor to the surface lattice of the PQDs with high binding energy (Figure 10b). The chelation effect suppresses the dynamic desorption, which in turn enhances the stability of CsPbI_3_ PQDs under various environmental factors [96]. Utilizing n-octylamine (OTAm, Figure 10a) and OA as ligands, the effective passivation of surface defect states can be achieved, resulting in a PLQY of up to 85.2%. With the increase in ligand concentration, the growth of colloidal PQDs is constrained, leading to a reduction in particle size. The decrease in particle size induces an enhancement of the quantum confinement effect, which contributes to the improvement in PLQY. Most importantly, it allows for large-scale production by 50 times (Figure 10c) [97].

#### 3.4.2. Alkylphosphines and Alkylphosphine Oxides

Alkyl phosphines and their oxides are also a class of L-type ligands. Wang et al. have demonstrated that treatment with trioctylphosphine (TOP, Figure 10d) can effectively restore the red luminescence of aged CsPbI_3_ PQDs immediately (Figure 10e) and can also significantly enhance the stability of PQDs against external environmental factors. This is mainly attributed to TOP’s ability to promote the migration of some ions on the surface of PQDs, to repair existing surface defects, and to prevent the formation of non-radiative recombination pathways. In addition, it has been found that other ligands, such as tributylphosphine (TBP) and triphenylphosphine (TPP), have similar effects on CsPbBr_3_ and CsPbI_3_ PQDs [98]. Li et al. have also confirmed that ligands such as TOP, TBP, and diphenylphosphane (DPP) significantly improve the tolerance of CsPbBr_3_ PQDs to external stimuli. More importantly, the modified PQD films exhibit superior optical performance and stability (Figure 10f). This is due to the large steric hindrance of the multi-branched phosphine ligands, which provide a dense protective layer for the PQDs [99]. The stability of CsPbX_3_ PQDs against ethanol treatment can be significantly improved by incorporating trioctylphosphine oxide (TOPO) into the OA/OAm system (Figure 10g). TOPO, through the strong coordination effect of its P=O group with the PQDs, firmly binds to the surface of the PQDs. Moreover, its highly branched structure provides a robust steric hindrance effect, preventing direct contact between ethanol molecules and the PQDs [100].

### 3.5. Z-Type Ligands

In inorganic ligands, K^+^ and Zn^2+^ are commonly used as Z-type ligands. CsPbI_3_ PQD films that have undergone K^+^ ligand exchange exhibit enhanced phase stability and a high PLQY of 96% [101]. After Zn^2+^ ligand exchange, CsPbBr_3_ PQDs can passivate uncoordinated sites and suppresses the nonradiative recombination of carriers within the PQDs [102]. The performance of various ligand-modified PQDs is presented in Table 1.

## 4. Summary and Outlook

In summary, we have reviewed factors contributing to the instability of inorganic lead halide (CsPbX_3_) PQDs, the solution-based ligand exchange methods, and the significant potential of various types of ligands in enhancing the luminescence performance and stability of PQDs. For ligand engineering, it is clear that factors such as the binding strength between ligands and PQDs, the chain length of the ligands, the polarity of the ligands, the steric effect of the ligands, and the concentration of the ligands all have a significant impact on the stability of PQDs. Ligands with strong binding groups can stably bind to the surface of PQDs, thereby maintaining their stability. There is an inverse relationship between the chain length and polarity of the ligands: higher polarity enhances binding with PQDs, but excessively high polarity may destroy the crystal structure and compromise stability. Hence, a balance between chain length and polarity is necessary. Multi-branched ligands, due to their steric effects, can reduce aggregation between PQDs and also provide a dense protective layer that prevents damage from oxygen and water molecules, thereby enhancing their stability. Additionally, the concentration of ligands affects the surface coverage of PQDs, which in turn influences their stability. By employing ligand engineering, the structural instability and environmental sensitivity issues of quantum dots have been successfully addressed, thereby paving the way for their broader application in optoelectronic devices. Meanwhile, the development of novel ligands with enhanced passivation and multifunctionality is necessary. Many challenges should be tackled by researchers, such as reducing the synthesis steps for ligands, enabling large-scale production of PQDs, and diversifying their practical applications. Additionally, investigating the mechanisms of the improvements in PL performance and stability will also be a key focus in future research.

## Figures and Tables

**Table 1:** Summary of the PLQY and stability of CsPbX_3_ QDs with various ligand modifications.

Type	Ligands	Methods *	PQDs	PLQY	Stability *	Ref.
X	DA	In situ	CsPbBr_3_	-	~94% (F *, in air, 28 d)	[49]
X	BOA	In situ	CsPbBr_3_	89%	PLQY: 84% (S *, ambient conditions, 90 d) PLQY: 81% (S, UV, 0.5 h)	[50]
X	CA	In situ	CsPbBr_3_	71%	80% (S, in water, 6 h) 75% (S, in ethanol, 6 h) 50% (S, UV, 6 h)	[51]
X	CTAB	In situ	CsPbBr_3_	70%	PLQY: 90% (S, ambient conditions, 7 d) 110% (-, UV, 13 h)	[52]
X	AA	Post	CsPbBr_3_	99%	72% (S, in air, 42 d) 33% (S, UV, 24 h)	[53]
X	AA	Post	CsPbI_3_	95%	No phase change (S, in air, 55 d) ~77% (S, UV, 4 h)	[53]
X	AA	Post	CsPbBrI_2_	95%	69% (S, in air, 42 d) 44% (S, UV, 24 h)	[53]
X	AA	Post	CsPb(Br/I)_3_ CsPbCl_1_._5_Br_1_._5_ CsPbClBr_2_	>95% 22% 51%	No exact numbers provided	[53]
X	ZW-PIMA-PEG	Post	CsPbBr_3_	70–80%	Complete loss of fluorescence (S, in polar solvents,1 month)	[54]
X	ZW-PIMA-OCA	Post	CsPbBr_3_	65–75%	No phase change (S, storage, 1.5 years) >85% (S, in acetone or ethanol, 8 months) Strong green fluorescence (P*, in water, 14 d)	[54]
X	BDGA	Post	CsPbBr_3_	~100%	~90% (S, ambient conditions, 60 d) ~95% (S, UV, 24 h) >80% (S, 60 °C, 1 h) No phase change (F, ambient conditions, 120 d)	[55]
X	DDAB	Post	CsPbBr_3_	~100%	PL emission is mainly preserved (S, in methanol/butanol, 7 months)	[62]
/	DDAB + NaSCN	Post	CsPbBr_3_	~100% (solution) 52% (films)	100% (-, UV, 1 h) 80% (S, in water, 1.5 h) 60% (F, heat to 200 °C)	[63]
/	DDAB + ZnBr_2_	Post	CsPbBr_3_	95%	85% (S, ambient conditions, 14 d) 90% (S, 50 °C, 60 min) 93% (S, UV, 24 h)	[67]
X	DDAF	Post	CsPbBr_3_	90%	No obvious change (S, heat to 100 °C)	[68]
X	OctBr	Post	CsPbCl_1_._5_Br_1_._5_	95%	80% (F, 380 K)	[69]
X	DTDB	In situ	CsPbBr_3_	~92%	80% (S, UV, 4.5 h) 80% (S, UV, 4.5 h) 95% (S, 80 °C, 4 h) 76% (S, in water, 17 h)	[70]
X	BBA	In situ	CsPbBr_3_	~86%	79% (S, in water, 72 h)	[73]
X	PFA	In situ	CsPbI_3_	>80%	80% (S, ambient conditions, 120 d)	[75]
/	ADA + ZnBr_2_	Post	CsPbBr_3_	~97%	93% (S, long-term stability, 65 d) Stronger brightness (P, in water, 15 min) 80% (S, 80 °C, 2 h)	[77]
X	PFGA	In situ	CsPbBr_3_	85%	~100% (S, long-term stability, 60 d)	[78]
X	OPA	In situ	CsPbI_3_	98%	50% (S, in air, 15 d) Better storage stability in nitrogen	[79]
X	TMPPA	In situ	CsPbBr_3_	~83%	86% (S, ambient conditions, 1.5 years)	[80]
X	DBSA	In situ	CsPbBr_3_	100%	PLQY: from 91.6% to 90.8% (S, 15 °C, 90 d) ~90% (S, UV, 1 h)	[84]
X	SDBS	In situ	CsPbI_3_	~91%	83% (S, ambient conditions, 60 d) Bright red luminescence (F, in water, 3 h) 72% (S, UV, 3 h)	[85]
X	DDT	Post	CsPbI_3_	90%	90% (S, UV, 3 d)	[86]
X	DDT	Post	CsPbI_3_	90%	PLQY: stable (S, ambient conditions, 15 d)	[87]
X	BET	Post	CsPbBr_3_	92%	>75% (S, ambient conditions, 10 d) >50% (F, ambient conditions, 15 d) >80% (S, UV, 1.5 h)	[89]
X	S-BET	Post	CsPbBr_3_	~100%	No exact numbers provided	[90]
X	PC	In situ	CsPbI_3_	~100%	Increased stability (S, in air, 6 months)	[91]
X	NKE-3	Post	CsPbBr_3_	∼25% (water dispersed)	29% (S, in water, 72 h)	[94]
X	NKE-12	Post	CsPbBr_3_	-	70% (S, in water, 14 d)	[95]
L	AHDA	In situ	CsPbI_3_	64.6%	PLQY: 63.7% (S, in air, 110 d) No phase change (F, 85 °C in air, 20 d) No phase change (F, UV, 500 min)	[96]
L	OTAm	In situ	CsPbBr_3_	~85%	>80% (S, 25 °C, 40% RH, 30 d)	[97]
L	TOP	Post	CsPbBr_1_._2_I_1_._8_	-	No change (S, in nitrogen, 14 d) 43% (S, heat from 20 to 90 °C) Increased PL intensity (S, UV, 12 h) No change (S, add ethanol)	[98]
L	TOP	In situ	CsPbBr_3_	83%	Bright (S, ambient conditions, 4 months) >50% (S, in ethanol, 30 h) ~70% (S, in water, 50 h) 90% (S, UV, 30 h) Bright (F, ambient conditions, 35 d)	[99]
L	DPP	In situ	CsPbBr_3_	81%	Bright (S, ambient conditions, 4 months) >50% (S, in ethanol, 30 h) ~90% (S, in water, 50 h) >80% (S, UV, 30 h) Bright (F, ambient conditions, 35 d)	[99]
L	TBP	In situ	CsPbBr_3_	75%	Bright (S, ambient conditions, 4 months) >20% (S, in ethanol, 30 h) ~70% (S, in water, 50 h) >80% (S, UV, 30 h)	[99]
L	TOPO	In situ	CsPbBr_3_	-	95% (S, in ethanol, 100 min)	[100]
Z	K^+^	Post	CsPbI_3_	96% (films)	No phase change (F, 25 °C, 40% RH, 2 months)	[101]
Z	Zn^2+^	Post	CsPbBr_3_	99%	-	[102]

* “Methods” refers to ligand engineering methods, including in situ ligand engineering and post-synthesis ligand engineering. * “Stability” refers to the remaining PL intensity, unless otherwise indicated. * In the table, the acronyms “S”, “F”, and “P” within the “Stability” column specifically denote solution, film, and powder, respectively.

## Data Availability

Not applicable.
